# Formation of Micro- and Nanostructures on the Nanotitanium Surface by Chemical Etching and Deposition of Titania Films by Atomic Layer Deposition (ALD)

**DOI:** 10.3390/ma8125460

**Published:** 2015-12-02

**Authors:** Denis V. Nazarov, Elena G. Zemtsova, Ruslan Z. Valiev, Vladimir M. Smirnov

**Affiliations:** Institute of Chemistry, Saint Petersburg State University, Universitetskii pr. 26, Saint Petersburg 198504, Russia; ezimtsova@yandex.ru (E.G.Z.); rzvaliev@mail.rb.ru (R.Z.V.); vms11@yandex.ru (V.M.S.)

**Keywords:** atomic layer deposition (ALD), chemical etching, nanostructured titanium, titanium implants, titania coatings

## Abstract

In this study, an integrated approach was used for the preparation of a nanotitanium-based bioactive material. The integrated approach included three methods: severe plastic deformation (SPD), chemical etching and atomic layer deposition (ALD). For the first time, it was experimentally shown that the nature of the etching medium (acidic or basic Piranha solutions) and the etching time have a significant qualitative impact on the nanotitanium surface structure both at the nano- and microscale. The etched samples were coated with crystalline biocompatible TiO_2_ films with a thickness of 20 nm by Atomic Layer Deposition (ALD). Comparative study of the adhesive and spreading properties of human osteoblasts MG-63 has demonstrated that presence of nano- and microscale structures and crystalline titanium oxide on the surface of nanotitanium improve bioactive properties of the material.

## 1. Introduction

Titanium and its alloys have a unique combination of acceptable mechanical properties and biocompatibility that allows it to be widely used in medical practice as a material for implants for the past 50 years [1,2,3,4]. Recently, interest to ultrafine-grained titanium (nanotitanium) in the field of biomedical materials was increased due to its excellent mechanical properties [5,6,7]. However, the mechanical properties are not the only characteristic that determines the success of the use of certain materials in the field of implantology. Other important characteristics of the material are its bioinertness, biocompatibility and bioactivity [1,2,3]. The transition to the production of materials based on nanotitanium and bioactive nanocoating is one of the backbone approaches to the synthesis of the new class of biomaterials that are widely used in modern dentistry, reconstructive surgery and orthopedics [2,3,4,8,9]. It should be noted that the improvement of the properties of the biomedical material requires the control of not only the chemical properties (primarily composition of the surface) but also surface properties (crystallinity, morphology and relief). Recent works showed that the presence of the structures at both micro- and nanoscale can give a significant contribution to the biocompatibility of the implants [10]. The micro- or nanoscale structures (developed relief) are usually achieved by machining, chemical or electrochemical etching [11,12].

It is currently generally recognized that the nanotitanium bioactivity can be improved by bioactive titanium dioxide coating [1,2,10,11,13]. Typically, for the production of coatings based on oxides, including preparation of titanium dioxide films, the following methods are used: anodic oxidation [14,15], plasma spraying [16,17], chemical vapor deposition [18], sol-gel [19,20], and others [11,19]. However, these methods in most cases do not allow obtaining a uniform coating on the developed relief surface and porous materials with preservation of their topography. One of the most acceptable and productive methods to create a continuous and uniform coating on the surface of such supports is the atomic layer deposition (ALD) [21].

ALD is based on the cyclic self-limiting gas-solid chemical reactions on the substrate surface whereby the film growth process takes place layer by layer [21]. While increasing number of surface chemical reaction (number of ALD cycles), the film thickness increases as well. In this way, the films of carbides, nitrides, sulfides, and metal film [22,23] can be obtained, but the method most widely used for the deposition of oxides [21,22,24,25,26]. ALD has two main advantages: high precision of the films thickness and high uniformity of the coating on the surface of planar and porous substrates [21,27,28]. Due to these features, ALD is widely used for the deposition of the films in the semiconductor industry [29], catalysis [30], solar energy [31], Li-ion batteries and supercapacitors [32]. Despite the advantages of the method, ALD has not yet found a wide application in the field of coatings for biomedical materials in general, and medical implants in particular. There are only a few papers devoted to the deposition of biocompatible coatings for implantology by ALD [13,33,34,35,36]. Undoubtedly this trend is very promising, and requires more detailed study.

In this paper, we demonstrate the opportunity of the integrated approach for creation of new implant material. The approach combines advantages of the methods described above. Severe plastic deformation (SPD) method improves mechanical properties of the material. Chemical etching produces a structure and morphology of the surface that is necessary for the successful and rapid engraftment of the implant. Finally, ALD method served for deposition of biocompatible and bioactive crystalline titania coating that protects implant from biological corrosion, keeps the etched titanium surface structure and favors the growth of bone tissue.

## 2. Experimental Section

### 2.1. Fabrication of Micro- and Nanoscale Structures on the Nanotitanium Surface by Controlled Liquid Phase Chemical Etching

Nanotitanium samples were prepared in Limited Liability Company “Nanomet”, Ufa, Russia, from titanium Grade 4. Titanium rods of 1 m length were subjected to Equal-Channel Angular Pressing by ECAP-Conform processing at 400 °C for 5 passes. The value of accumulated true strain was 3.5. Detailed description of ECAP-Conform processing technique can be found in [37]. After ECAP-Conform processing, the billets were subjected to drawing at 200 °C resulting in production of rods with a diameter of 6 mm. The average grain size of nanotitanium was ~50–100 nm. Samples before etching were treated by machining. Firstly, nanotitanium rod was cut into discs having thickness of 2–3 mm by the Buehler IsoMet 1000 machine (Buehler, Lake Bluff, IL, USA). Then, these discs were ground and polished by semiautomatic machine Buehler MiniMet 1000 (Buehler, Lake Bluff, IL, USA) to mirror-like surface (roughness less than 5 nm). Then the nanostructured titanium discs were cleaned in ultrasonic bath in acetone and deionized water for 15 min. Finally the samples were dropped into a basic (NH_4_OH/H_2_O_2_) or acidic (H_2_SO_4_/H_2_O_2_) Piranha solution at room temperature. The ratio of reactants was 7/3; exposure times were 15 min, 2 and 24 h for both types of etching media. Immediately after etching the samples were taken out of the etchant and thoroughly washed in distilled water and acetone using an ultrasonic bath.

### 2.2. Synthesis of Titanium Oxide Nanostructures by ALD on the Surface of Chemically Etched Nanotitanium

Titanium oxide coatings were obtained by ALD on the surface of the polished, etched nanotitanium and monocrystalline silicon plates (100) that act as a witness for ellipsometry and X-ray reflectometry (XRR) measurements. The deposition was performed at the commercial “Nanoserf” setup (Nanoengineering Ltd., St. Petersburg, Russia) in the Resource Center “Innovative technologies of composite nanomaterials” Saint Petersburg State University (SPbSU). The deposition was performed in hot-wall, flow-type reactor having slot-type geometry. Base pressure of the reactor is 6.5 Pa. Nitrogen with purity of 99.9999% was used as carrier and purging gas. Titanium isopropoxide (Sigma Aldrich, St. Louis, MO, USA, 99.999%) was supplied into a reaction chamber as a solution in isooctane by the injection valve. Isooctane acted as a carrier gas, which evaporates quickly after injection into the hot reactor and due to isooctane chemical properties it does not cause adverse chemical reactions. Volume ratio of the titanium isopropoxide/isooctane was 1/20. Flowrate of the titanium isopropoxide for one cycle was about 4 μmol, and the pulse duration was 20 ms. Water source was delivered by vapor with the water ampoule being held at 27 °C, pulse duration was 500 ms. Deposition was carried out as follows: Before precursor pulse the supply of inert gas was stopped and the reactor was evacuated for 5 s to a pressure of about 0.7 hPa. Then reagent was pulsed and held without pumping and purging for 0.5 s (static mode). Since the vapor pressure of the reagent considerably higher than the pressure inside the reactor (0.7 hPa), the precursor expands and occupies all the volume of the reactor. After that, pumping occurred within 5 s to remove the reaction products and excess reactants. Then, a purge pulse proceeded at a pressure of 25 hPa for further removal of reagents during 20 s. Thus, the overall purge stage lasted 30 s. Reactor pressure at water and titanium isopropoxide solution pulse increased to 30 and 45 hPa respectively. The temperature of the reactor chamber was maintained at 250 °C. Total reactor gas flow at purge pulse was 300 sccm (standard cubic centimeters per minute). Applied number of ALD cycles was 400 and thickness was 20 nm, which corresponds to TiO_2_ growth rate 0.05 nm/cycle.

### 2.3. The Study of Morphology, Relief, Structure and Composition

The thickness of TiO_2_ film deposited on the surface of silicon witness and polished non-etched nanotitanium was determined by the spectral ellipsometry and X-ray reflectometry (XRR). The ellipsometry instrument (Ellips-1891 SAG, Novosibirsk, Russia) provided an accuracy of the film thickness determination of 0.3 nm in the thickness range of 1–100 nm. X-ray reflectometry (XRR) and X-ray diffraction studies (XRD) in both symmetrical θ/2θ and asymmetrical surface sensitive grazing incidence (GID) modes were performed using a Bruker D8 DISCOVER high-resolution diffractometer (Bruker, Billerica, MA, USA) and analyzed with TOPAS 4.2 (XRD) and LEPTOS 7.7 (XRR) software at the Resource Centre “X-ray Diffraction Studies” SPbSU.

The topography of the samples surfaces was studied using a Solver P47 Pro (NT-MDT, Moscow, Russia) probe microscope in the tapping mode via atomic force microscopy (AFM). The AFM study was conducted at 3–4 points on the surface of the sample. Scanning electron microphotographs were obtained using the SEM Zeiss Merlin at the “Nanotechnology” Interdisciplinary Resource Centre SPbSU. Microscope spatial resolution was of around 1 nm and magnification up to 200 k×. In-lens SE and SE2 regimes was used.

X-ray photoelectron spectra were registered with a “Thermo Fisher Scientific Escalab 250Xi” spectrometer (Thermo Fisher, Waltham, MA, USA) at the Resource Centre of “Physical Methods of Surface Investigation” SPbSU. The samples were excited by Al Kα (1486.7 eV) X-rays in a vacuum of 7 × 10^−8^ Pa. The sample charging was automatically compensated. The binding energy scale has been referenced using the C1s carbon line (284.8 eV) [38].

### 2.4. Adhesive and Spreading Properties of the Human Osteoblasts MG-63 on the Titania Nanostructured Surface

Adhesion properties and the rate of the cells growth of the human osteoblasts have been evaluated using scanning electron microscopy (SEM). For this purpose, titanium samples treated by the cells have been used. The study is performed in the Institute of Cytology (RAS, St. Petersburg, Russia). The following samples were investigated:

Sample 1—Polished coarse grained titanium; Sample 2—Polished nanotitanium; Sample 3—Nanotitanium etched in basic Piranha for 15 min; Sample 4—Nanotitanium etched in basic Piranha for 15 min with subsequent deposition of titania (thickness of the layer is 20 nm); Sample 5—Nanotitanium etched in acid Piranha for 15 min; and Sample 6—Nanotitanium etched in acid Piranha for 15 min with subsequent deposition of titania (thickness of the layer is 20 nm).

All the samples have been placed into the Petri dishes and sterilized by means of ozonation. Cell line MG-63 has been spread onto the surface from 100 μL nutrient solution in the manner that the non-flowing drop is formed. The cell concentration was 1 × 10^5^/cm^2^ of the sample. The samples so coated with cells suspension are treated in the conditions of СО_2_-incubator at 37 °C for 3 h. This time is supposed to be enough for the cells adhesion on the samples surfaces. After that, nutrient solution has been added to the Petri dishes. In the control experiment, cell suspension has been spread onto the surface of the cultural dish.

After 5 days of cultivation, nutrient solution has been removed, washed three times with phosphate buffer saline (PBS) and fixed in the 20-fold volume of 2.5% solution of glutaric aldehyde. Scanning electron microscopy (JSM-35.7, Tokyo, Japan) has been used for the evaluation of the state of cells (*i.e.*, the adhesion and spreading modes of the cells on the samples surfaces).

## 3. Results and Discussion

### 3.1. Chemical Etching, Composition, Morphology and Surface Relief

According to [11,39] the surface topography of the titanium greatly varies during the etching process. The morphology mainly depends on the nature of the etchant. Acidic etchant (sulfuric or fluorosulfonic acid) forms spongy structure, processing of trifluoroacetic acid results in the formation of large (up to microns in a diameter and 100 nm in depth) craters, whereas alkaline etchants (aqueous ammonia) gives the network structure, the mesh size ranges depending on the concentration of the etchant [39]. The use of the chemical etching method for controlling the surface roughness is described in more detail in reviews [11,40]. However, the information on the etched nanostructured titanium has almost not represented until now. Thus, the first step of the work was a brief study of the effect of the nature of etchant and etching time on the relief, structure and surface morphology of the nanotitanium.

As etchants we choose the acidic (H_2_SO_4_/H_2_O_2_) and basic (NH_4_OH/H_2_O_2_) Piranha solutions. These solutions are widely used for cleaning and hydrophilization of the surface of various materials and with respect to metal materials and particularly for titanium this solution can act as etchants [39]. Selecting the regime of etching (time and concentration of etchant) we were guided by the data for coarse grained titanium [39], as well as our previous studies devoted to etching of the nanotitanium in HF and HF + HNO_3_ solutions [41].

**Figure 1:**
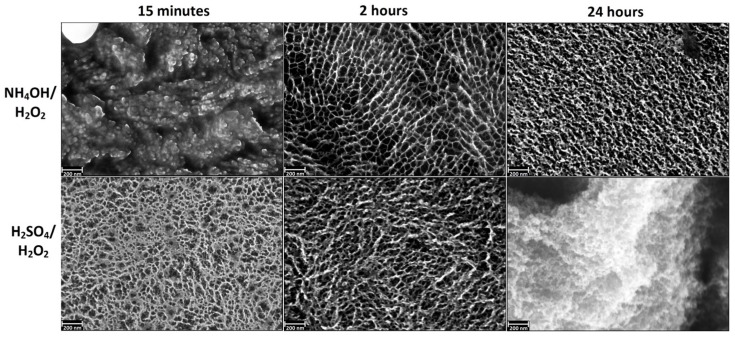
Characteristic scanning electron microscopy (SEM) images of nanotitanium etched in NH_4_OH/H_2_O_2_ and H_2_SO_4_/H_2_O_2_ solutions during 15 min, 2 h and 24 h (magnification: 200 k×, scale bar: 200 nm).

Figure 1 shows the SEM images (magnification of 200 k×) of the samples after etching in acidic and alkaline Piranha solutions at exposures of 15 min, 2 h and 24 h. SEM data indicate that the etching in the acidic Piranha in the range from 15 min to a few hours leads to “sponge” nanoscale structure, which is destroyed at longer exposures (about 24 h). The formation of such structures is also characteristic of coarse grained titanium [39]. Alkaline Piranha solution acts in the different manner; the sample after a 15 min treatment is characterized by “coral” structure. The appearance of “coral” structure is likely to be caused by etching in the first place on the grain boundaries. Thus, the result of this etching is the appearance of grains of nanostructured titanium. By increasing the etching time to 2 h, etching character is changed. In this case, firstly nanotitanium grains are etched and the grain boundaries only remain on surface of the sample. Changes in the nature of etching are likely due to the gradual oxidation and passivation of the titanium grain boundaries. Thus, the 2 h etching results in a “mesh” structure of the surface, which has the size of the grid comparable to nanotitanium grain size (50–100 nm). A further increase in etching time leads to the formation of a disordered “sponge” structure.

**Figure 2:**
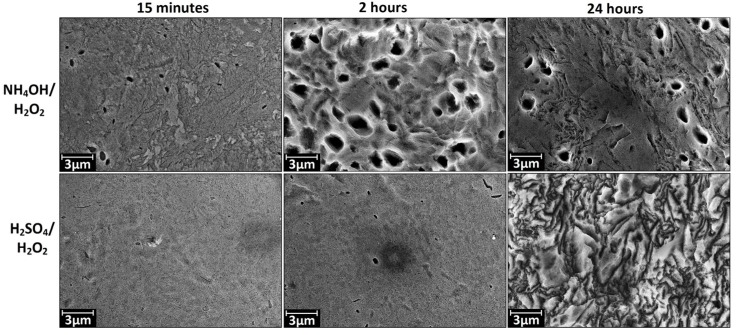
Characteristic SEM images of nanotitanium etched in NH_4_OH/H_2_O_2_ and H_2_SO_4_/H_2_O_2_ solutions during 15 min, 2 h and 24 h (magnification: 10 k×, scale bar: 3 μm).

The SEM data at magnification of 10 k× (Figure 2) shows that the etching in H_2_SO_4_/H_2_O_2_ leads to noticeable changes in morphology on microscale at exposure time close to 24 h. The changes manifest themselves in the presence of randomly distributed structures with irregularly shape. Etching in NH_4_OH/H_2_O_2_ leads to the formation of the pits with a diameter of 1–2 μm. A marked degree of this microscale structures is observed when expose time is more than 15 min and it reaches the maximum number when exposing time is about 2 h.

**Figure 3:**
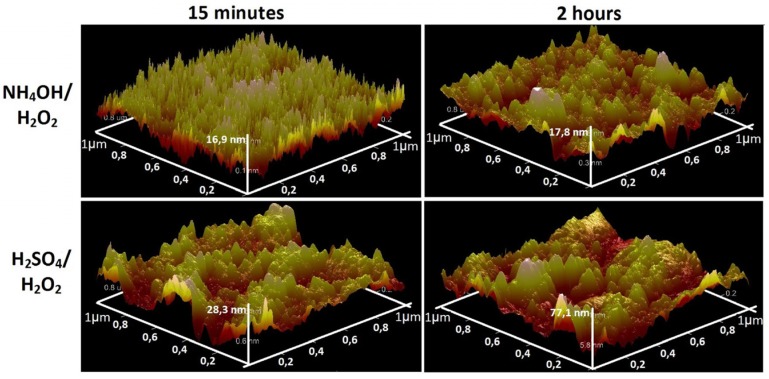
Atomic force microscopy (AFM) topographies (1 × 1 µm^2^) of the surface of nanotitanium etched in NH_4_OH/H_2_O_2_ and H_2_SO_4_/H_2_O_2_ solutions during 15 min and 2 h.

**Figure 4:**
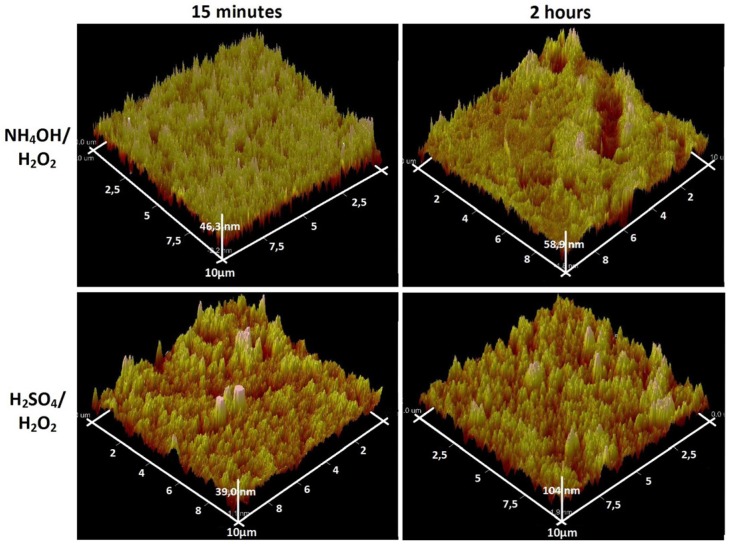
AFM topographies (10 × 10 µm^2^) of the surface of nanotitanium etched in NH_4_OH/H_2_O_2_ and H_2_SO_4_/H_2_O_2_ solutions during 15 min and 2 h.

Analysis of the AFM data on the scale of 1 × 1 μm^2^ (Figure 3) and 10 × 10 μm^2^ (Figure 4) showed a significant difference the surface topography of the sample NH_4_OH/H_2_O_2_-15 min etching (“coral” structure) from the rest. The values of the mean-square roughness (RMS) naturally increase with increasing exposure time (from 15 min to 24 h). This values increase from 3.9 to 29 nm for H_2_SO_4_/H_2_O_2_ and from 7.3 to 9.2 nm for NH_4_OH/H_2_O_2_. RMS of the initial polished nanotitanium equals to 2.6.

In summary, the SEM and AFM data show opportunity for creation of the developed relief on the nanotitanium surface by chemical etching in Piranha solutions. It is worth noting that the relief has various type of morphology (“coral”, “mesh”, “sponge”, “micropits”) both at nano- and microscale.

**Figure 5:**
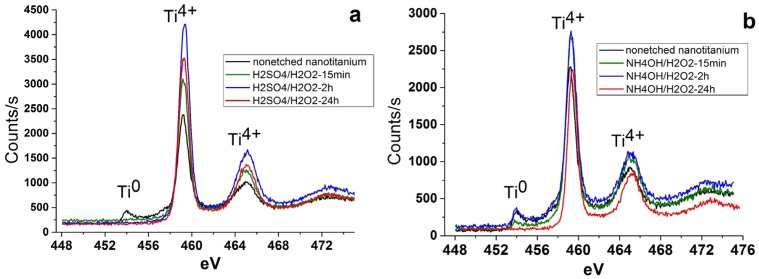
X-ray photoelectron Ti2p level spectra of nanotitanium etched in (**a**) acidic Piranha and (**b**) basic Piranha.

The study of the composition of the surface of the etched samples by X-ray photoelectron spectroscopy (XPS) showed the presence of Ti, O and C. The carbon contamination seemingly is caused by adventitious atmospheric hydrocarbon on the surface of the samples [38]. No other contaminants of the samples surface were found. It should be noted that the peak of Ti^0^ (453.9 eV) reduces or disappears after etching (Figure 5). This fact indicates that the oxidation accompanies the etching.

### 3.2. Deposition of TiO_2_ Nanocoatings, Structure, Composition and Morphology

It is known [11,12] that the natural oxides and oxides produced during etching cannot fully prevent the implant material from corrosion in aggressive biological environment. Thus, additional protective layers should be deposited on the implant surface. It is to be noted that the degree of crystallinity surface oxide layer of the titanium implants significantly affects on engraftment of bone tissue [13]. Higher crystallinity leads to more favorable growth of hydroxyapatite (the main inorganic component of bone tissue) [42]. Rutile exhibits the most suitable crystalline structure, however, it is seen from experimental data that rutile does not demonstrate significant advantage at *in vitro* medical experiments in comparison to anatase [43]. On the contrary, anatase film enhances osteoblast adhesion, spreading and proliferation by affecting surface contact angles and/or wettability [42].

**Figure 6:**
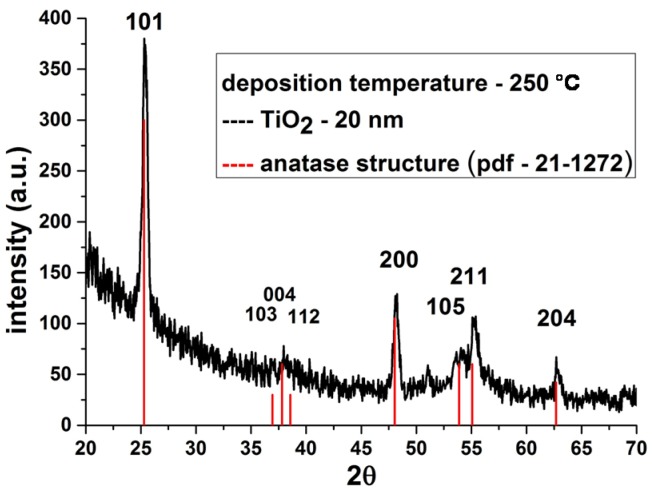
X-ray diffraction studies (XRD) patterns of TiO_2_ coatings deposited on titanium substrates at 250 °C.

In connection with the above, an important task for our research was to produce polycrystalline films of titanium dioxide. The main parameters that influence on the film crystallinity are the deposition temperature and the layer thickness. According to the literature data [22] crystalline films are obtained at temperatures of 225 °C and above, but thickness of the film/number of ALD cycles is 12 nm/300 cycles [13]. In this regard, nanofilms of crystalline titanium dioxide on the surface of the etched nanotitanium were synthesized at a temperature of 250 °C for 400 cycles. Indeed, preliminary deposition showed that the films obtained after 400 ALD cycles and at a temperature 250 °C are polycrystalline and have the anatase structure (Figure 6).

Based on the SEM and AFM data, we chose the following samples for TiO_2_ deposition:

(1) NH_4_OH/H_2_O_2_-15 min—“coral” structure.
(2) NH_4_OH/H_2_O_2_-2 h—“mesh” structure.
(3) H_2_SO_4_/H_2_O_2_-15 min—“sponge” structure.
(4) H_2_SO_4_/H_2_O_2_-24 h—disordered, developed relief.

Since the determination of the thickness of the films on porous objects by spectroscopic ellipsometry and XRR methods is almost impossible the silicon wafer (the witness) was placed in the reactor near to the titanium samples. Spectroscopic ellipsometry and XRR showed the TiO_2_ film thickness of about 20 nm.

**Figure 7:**
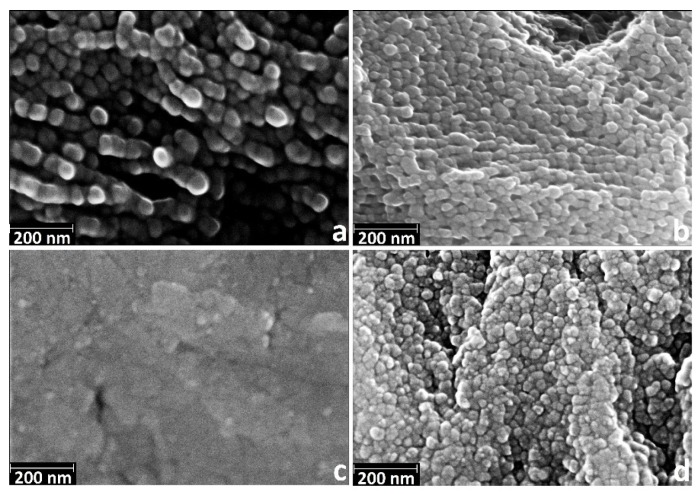
Characteristic SEM images of nanotitanium etched in Piranha solutions and TiO_2_ coated by ALD (magnification: 200 k×, scale bar: 200 nm) (**a**) NH_4_OH/H_2_O_2_-15 min; (**b**) NH_4_OH/H_2_O_2_-2 h; (**c**) H_2_SO_4_/H_2_O_2_-15 min; and (**d**) H_2_SO_4_/H_2_O_2_-24 h.

The SEM data (Figure 7) suggest that nanoscale relief is saved only for the sample NH_4_OH/H_2_O_2_-15 min (Figure 7a)—“coral” structure. This fact is quite expected, as the size of the nanostructures after etching does not exceed a few tens of nanometers. Thus, after the deposition of films, the nanostructures are overgrown by layer of titanium dioxide.

In general, all the samples after deposition demonstrate the presence of the grains of 30–100 nm in diameter. It is worth noting that grains packing of nanotitanium etched in NH_4_OH/H_2_O_2_ is much higher after 2 h than after 15 min. For the samples after H_2_SO_4_/H_2_O_2_ etching, grains appear with the increased etching time.

**Figure 8:**
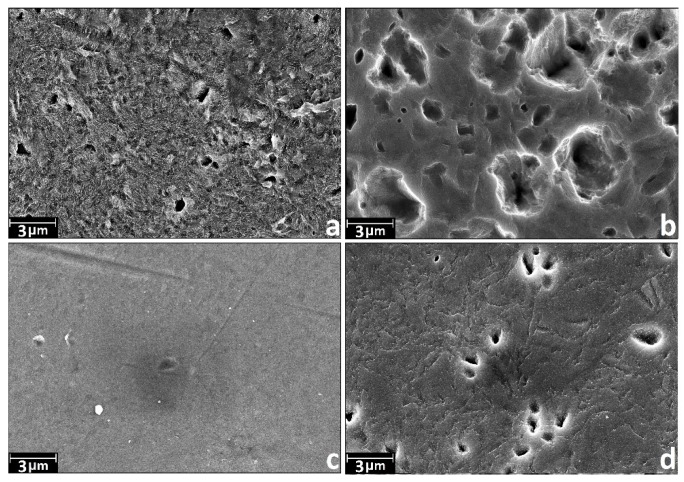
Characteristic SEM images of nanotitanium etched in Piranha solutions and TiO_2_ coated by atomic layer deposition (ALD) (magnification: 10 k×, scale bar: 3 μm) (**a**) NH_4_OH/H_2_O_2_-15 min; (**b**) NH_4_OH/H_2_O_2_-2 h; (**c**) H_2_SO_4_/H_2_O_2_-15 min; and (**d**) H_2_SO_4_/H_2_O_2_-24 h.

Note that the coating preserves initial relief of the etched nanotitanium at the micro scale. However, we found some healing of micrometer holes that is indicative for the sample NH_4_OH/H_2_O_2_-2 h (Figure 8b) and, oppositely, appearance of such holes for H_2_SO_4_/H_2_O_2_-24 h (Figure 8d).

**Figure 9:**
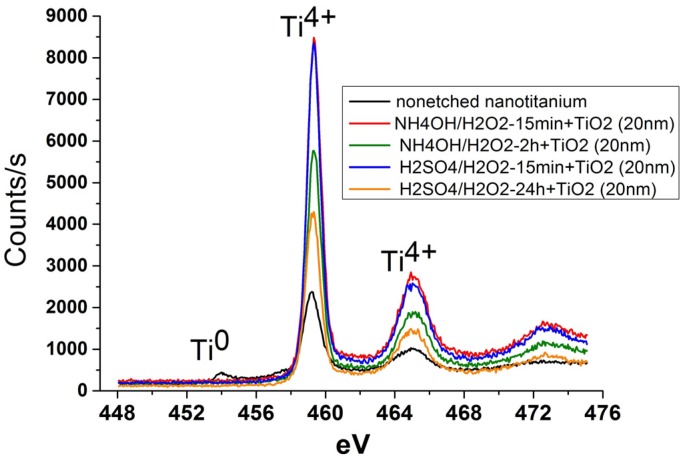
X-ray photoelectron Ti2p level spectra of nanotitanium etched in Piranha solutions and TiO_2_ coated by ALD.

The study of the surface composition of TiO_2_ coated samples by XPS showed only the presence of Ti, O and С. Note that the complete disappearance of the Ti^0^ peaks is observed for all films (Figure 9). This observation confirms the full coverage of the surface of the sample by continuous TiO_2_ layer.

### 3.3. Adhesive and Spreading Properties of the Human Osteoblasts MG-63

**Figure 10:**
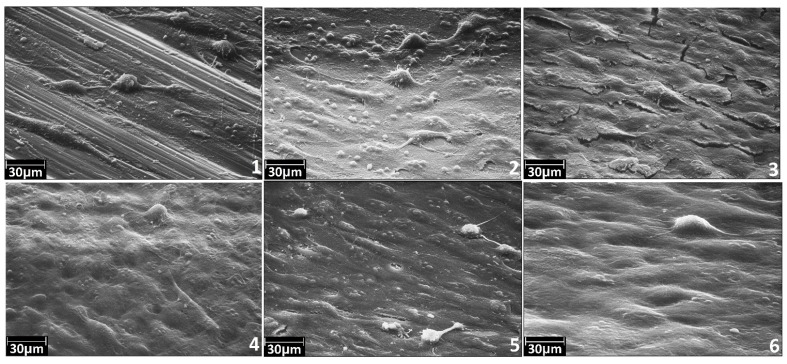
Characteristic SEM images for the samples No. 1–6 after adhesion and spreading of the human osteoblasts MG-63: (**1**) polished coarse grained titanium; (**2**) polished nanotitanium; (**3**) nanotitanium etched in NH_4_OH/H_2_O_2_-15 min; (**4**) nanotitanium etched in NH_4_OH/H_2_O_2_-15 min and TiO_2_ coated by ALD; (**5**) nanotitanium etched in H_2_SO_4_/H_2_O_2_-15 min; and (**6**) nanotitanium etched in H_2_SO_4_/H_2_O_2_-15 min and TiO_2_ coated by ALD.

To investigate the biocompatibility of biomedical implant surfaces, a number of analytical methods exist to assess cellular responses. The methods include the study of the cell adhesion, proliferation, migration, differentiation and cell survival. Adhesion and spreading are the initial and necessary steps in the process of bone healing. Thus, adhesive and spreading properties give initial information on biocompatibility and bioactivity of the implant. We applied these methods to study cellular responses. Results of the study are presented at Figure 10.

Despite the fact that the cells concentration was uniform while coating the samples, the surface of Sample 1 (coarse grained titanium) contains single cells. It can indicate low adhesion properties of this sample for the studied cell line. For the Sample 2 (nanotitanium), adhesion is stronger for this kind of cells. The more adhesive surface for this cells type is found for the nanotitanium sample etched in basic Piranha for 15 min (Sample 3) as well as one etched in acid Piranha for 15 min (Sample 5).

The nanotitanium etched and subsequently coated by 20 nm titania layers (Samples 4 and 6) possesses the most adhesive surface for this cells type. The cells form the uniform monolayer, without clear morphology changes.

It can be preliminarily summarized from the SEM experiments that the most adhesive surface for cultivation of cell line MG-63 is formed from etched nanotitanium subsequently covered by 20 nm TiO_2_ layer.

## 4. Conclusions

In this work, we have demonstrated the opportunity of preparation of a nanotitanium-based bioactive material using integrated approach. The integrated approach included three methods: severe plastic deformation (SPD), chemical etching and atomic layer deposition (ALD).

For the first time, it has been shown experimentally that the nature of the etchant and the etching time have a significant impact on the nanotitanium surface morphology at nano- and microscale. The nanoscale structures such as “mesh”, “sponge”, “coral” or “disordered, developed relief” can be obtained by chemical etching using various type of etchant (acidic or basic Piranha solutions) and exposure time. At the microscale, the pits can be obtained with the diameter 1–2 μm.

On the surface of these structures, crystalline coating of titanium oxide about 20 nm thickness has been successfully deposited by ALD. It was shown that after coating the relief of samples is completely retained at the microscale and only partially at the nanoscale. The data on evaluation of adhesive and spreading properties of the human osteoblasts MG-63 cell culture showed that the samples etching in both Piranha solution and subsequently coated with TiO_2_ films have the much better adhesive and spreading properties than untreated ones. Thus, it was shown that integrated approach is available to prepare biocompatible and bioactive material.
